# Osteogenic Cell Behavior on Titanium Surfaces in Hard Tissue

**DOI:** 10.3390/jcm8050604

**Published:** 2019-05-02

**Authors:** Jung-Yoo Choi, Tomas Albrektsson, Young-Jun Jeon, In-Sung Luke Yeo

**Affiliations:** 1 Dental Research Institute, Seoul National University, Seoul 03080, Korea; jychoi55@snu.ac.kr (J.-Y.C.); yoowjs@snu.ac.kr (Y.-J.J.); 2 Department of Biomaterials, Sahlgrenska Academy, University of Gothenburg, 40530 Gothenburg, Sweden; tomas.albrektsson@biomaterials.gu.se; 3 Department of Prosthodontics, Faculty of Odontology, Malmö University, 21118 Malmö, Sweden; 4 Department of Prosthodontics, School of Dentistry and Dental Research Institute, Seoul National University, 101 Daehak-ro, Jongro-gu, Seoul 03080, Korea

**Keywords:** osteogenesis, cell plasticity, dental implants, electron microscopy, scanning transmission electron microscopy, bone-implant interface

## Abstract

It is challenging to remove dental implants once they have been inserted into the bone because it is hard to visualize the actual process of bone formation after implant installation, not to mention the cellular events that occur therein. During bone formation, contact osteogenesis occurs on roughened implant surfaces, while distance osteogenesis occurs on smooth implant surfaces. In the literature, there have been many in vitro model studies of bone formation on simulated dental implants using flattened titanium (Ti) discs; however, the purpose of this study was to identify the in vivo cell responses to the implant surfaces on actual, three-dimensional (3D) dental Ti implants and the surrounding bone in contact with such implants at the electron microscopic level using two different types of implant surfaces. In particular, the different parts of the implant structures were scrutinized. In this study, dental implants were installed in rabbit tibiae. The implants and bone were removed on day 10 and, subsequently, assessed using scanning electron microscopy (SEM), immunofluorescence microscopy (IF), transmission electron microscopy (TEM), focused ion-beam (FIB) system with Cs-corrected TEM (Cs-STEM), and confocal laser scanning microscopy (CLSM)—which were used to determine the implant surface characteristics and to identify the cells according to the different structural parts of the turned and roughened implants. The cell attachment pattern was revealed according to the different structural components of each implant surface and bone. Different cell responses to the implant surfaces and the surrounding bone were attained at an electron microscopic level in an in vivo model. These results shed light on cell behavioral patterns that occur during bone regeneration and could be a guide in the use of electron microscopy for 3D dental implants in an in vivo model.

## 1. Introduction

Dental implants are cylindrical prosthetics with screw threads, usually made of titanium (Ti), which are used to replace missing teeth and to support the mastication function of artificial teeth. However, the biological contact with the surface of dental implants is different from that with natural teeth. Osseointegration, the direct contact between bone and implant, is viewed as a hard tissue encapsulation, a foreign body immune reaction that isolates the implant; this bone response is generally accepted as a bio-affinitive reaction to a biocompatible material [1]. To enhance the activity of osteogenic cells in bone integration, the physical and chemical characteristics of the implant surface—including the surface energy, wettability, and topography—are modified, because direct enhancement of the bone surface is much more difficult [2,3,4,5,6,7,8,9,10,11]. Such surface treatments can be, in reality, an enhancement to encase the foreign body in hard connective tissue [1,12,13]. Therefore, it is necessary to investigate the in vivo biological response to implant surfaces at the cellular level.

To control the variables, and thereby produce a sound scientific result, in vitro studies using purified cell lines and flat Ti discs with modified surfaces can be performed. However, promising in vitro results in cell responses to such Ti discs do not guarantee obtainment of the desired reactions for Ti implants with the same modified surfaces in in vivo environments. The Ti implants used today to treat patients are screw-shaped, rather than flat disc-shaped. Screw threads have macro- and microstructures—such as roots, flanks, and crests—which the homogeneous Ti disc surfaces for the in vitro experiments are unable to simulate [14]. The cell lines for in vitro tests are usually osteoblast-like cells, rather than human osteogenic cells, and the in vivo environment is very different from an in vitro cell culture medium [5,8,15]. Nonetheless, osteoblastic cell lines in in vitro tests form a simplified system which does not take into account aspects such as immune responses [16]. Therefore, translational evidence is required to create a bridge between the in vitro cell results and the in vivo tissue results—that is, the cellular response to a Ti implant surface in the in vivo environment.

This study aimed to observe Ti implants and the surrounding bone in contact with such implants at the electron microscopic level to identify the in vivo cell responses to the implant surfaces

## 2. Materials and Methods

### 2.1. In Vivo Study

**Figure 1:**
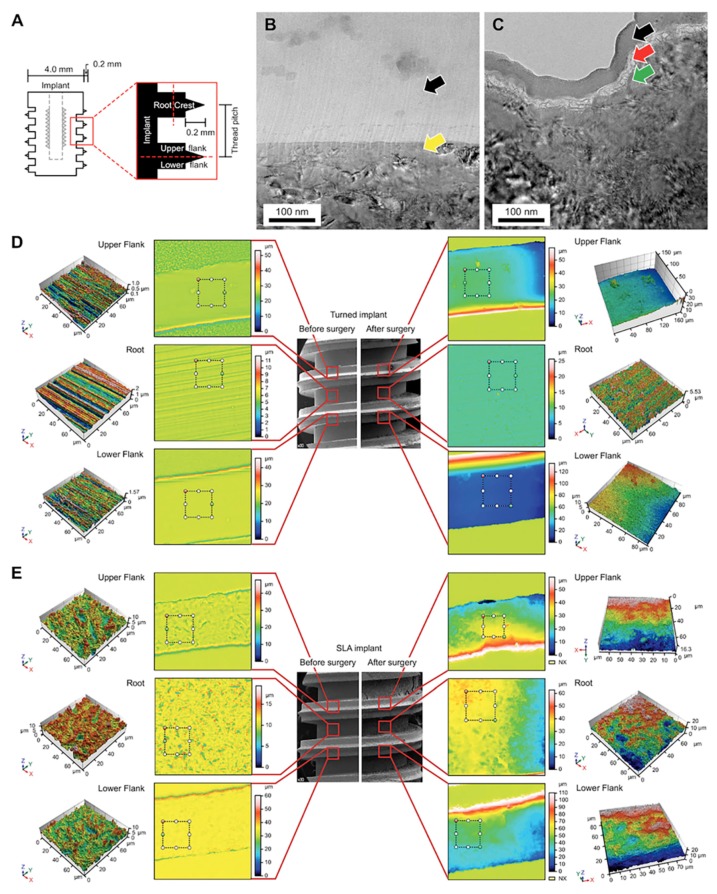
(**A**) Simplified diagram of, and terminology regarding, the screw-shaped implants used in this study. The inner half, close to the minor diameter of the implant, was defined as the root area. The outer half, close to the major diameter of the implant, was called the crest area. The upper half of the thread was defined as the upper flank (UF), and the lower half was the lower flank (LF). (**B**) Cs-corrected transmission electron microscopy (Cs-STEM) analysis retrieved from focused ion beam specimens of the turned and (**C**) acid-etched (SLA) implants on day 10. There were no cells detected on the turned surface (yellow arrow) beneath the Pt-coated layer (black arrow), whereas, cells were detected on SLA surface (red arrow). (**D**) Confocal laser scanning microscopy (CLSM) of the turned and (**E**) SLA surfaces measured in root, UF, and LF. The turned implant revealed a smooth texture, and no cells were seen after in vivo experiment. The SLA implants displayed cell attachment in the root area, depicted as irregular structure of grey color on top of roughened topography in the 3D mapping of the CLSM.

This study was approved by the Ethics Committee of the Animal Experimentation of the Institutional Animal Care and Use Committee (CRONEX-IACUC 201702003; Cronex, Hwasung, Korea). All experiments were conducted in accordance with the ARRIVE guidelines for reporting in vivo animal experiments [17]. A total of 8 male New Zealand white rabbits (age: 1–2 years; body weight: 2.6–3.0 kg) with no signs of disease were used. The rabbits were anaesthetized via intramuscular injection of tiletamine/zolazepam (15 mg/kg; Zoletil 50, Virbac Korea Co., Ltd., Seoul, Korea) and xylazine (5 mg/kg; Rompun, Bayer Korea Ltd., Seoul, Korea). Before surgery, the skin over the area of the proximal tibia was shaved and washed with betadine, and an antibiotic (Cefazolin, Yuhan Co., Seoul, Korea) was intramuscularly administered. Lidocaine was locally injected into each surgical site. The skin was incised, and the tibiae were exposed after muscle dissection and periosteal elevation. Drills and profuse sterile saline irrigation were used to prepare the implant sites on the flat tibial surface. The drilling was performed with a final diameter of 4.0 mm at the upper cortical bone, in which the implants were installed in cortical bone and medullary space. Only the V-shaped parts of the threads were engaged (Figure 1A). A total of 5 rabbits received acid-etched (SLA) implants only. Each rabbit received 4 SLA implants, 2 on each side of the rabbit tibia. Three rabbits received turned implants only, each receiving 4 turned implants, 2 on each side of the tibia. The cover screw was covered. The muscle and fascia were sutured with absorbable 4–0 Vicryl sutures, and the outer dermis was closed with a nylon suture. The rabbits were separately housed after surgery. All rabbits were sacrificed via an intravenous overdose of potassium chloride after 10 days of bone healing. After 10 days [1,18,19], the tibiae were exposed, all of the inserted implants were removed through unscrewing, and the surrounding bone was surgically removed en bloc with an adjacent bone collar and immediately placed in Karnovsky’s solution for cell fixation in falcon tubes, while the specimens for fluorescence immunocytochemistry were preserved in Roswell Park Memorial Institute (RPMI) media and fetal bovine serum (Gibco, Thermo Fisher Scientific, Waltham, MA, USA) in cell culture dish.

### 2.2. Sample Preparation and Implant Surface Modification

Herein, 26 Ti sandblasted, large-grit, and SLA implants and 18 turned implants were used (Deep Implant System, Inc., Seongnam, Korea). The implants were made of grade 4 commercially pure Ti by computer numerical control (CNC) machining. The implant surface was called ‘turned’ when the surface had no further modification after CNC machining. The SLA surface was made by sandblasting the implant surface with 250–500 μm alumina particles and by etching the surface with HCl/H_2_SO_4_ acid mixture. All of the implants were 4.0 mm in diameter and 5.0 mm in length. A total of 20 SLA implants were used in an in vivo study, and 6 were used in the surface analysis, while 12 turned implants were used in the in vivo analysis, and 6 were used in the surface analysis.

### 2.3. Surface Characteristics

Among the 6 SLA implants and 6 turned implants used in the surface analysis, 2 of each type of implant were used for scanning electron microscopy (SEM; Hitachi S-4700, Hitachi, Tokyo, Japan), 2 were used for confocal laser scanning microscopy (CLSM; LSM 800, Carl Zeiss AG, Oberkochen, Germany), and the remaining 2 implants were used for focused ion beam (FIB; Helios 650, FEI, Hillsboro, OR, USA) and Cs-corrected transmission electron microscopy (Cs-STEM; JEM-ARM200F, Cold FEG, FEOL Ltd., Tokyo, Japan), which are capable of producing transmission electron microscopy (TEM) images directly from an undecalcified specimen. SEM was used to observe the topographical features, while CLSM was used to analyze the surface roughness levels. The measured area roughness parameters included the average height deviation value (S_a_) and the developed surface area ratio (S_dr_). FIB and Cs-STEM were used to observe the undecalcified implant surface directly without any resin embedding.

### 2.4. Scanning Electron Microscopy (SEM) Analysis

The retrieved implant specimens and surrounding bony specimens were fixed with Karnovsky’s solution and washed in 0.1 M phosphate buffer saline (PBS) 3 times every 15 min. The specimens were dehydrated through a graded 70–100% ethanol series and then treated with hexamethyldisilazane for 15 min. The surrounding bone specimens were cut in half around the round hole in which the implant had been inserted, after degradation with 80% ethanol using rotary discs within an appropriate amount of time. Prior to the SEM analysis, the implant and bone specimens were sputter coated with a thin film of platinum to protect the implant and bony surfaces. All specimens were handled with Ti forceps and surgical gloves in a clean laboratory environment. Each implant and bone sample was attached using adhesive carbon tape, as well as aluminum tape, on the SEM sample stub. The samples were inserted into a Hitachi S-4700 (Hitachi, Tokyo, Japan), which was operated at 20 kV.

### 2.5. Immunofluorescence Microscopy (IF) Analysis

Prior to the sacrifice of the rabbit tibiae, the implants were removed from each tibia and, along with the surrounding bone, the specimens were preserved for 3 h in the refrigerator in the RPMI media, which contained penicillin (50 U/mL) and streptomycin (50 μg/mL). The cells underwent immunostaining and were incubated for 15 min with a protein block (DAKO, Agilent, Santa Clara, CA, USA, X0909) to remove non-specific binding protein. The cells were then incubated for 30 min with a diluted osteocalcin primary antibody (1:100 dilution in 3% bovine serum albumin (BSA), #MA120788, Thermo Fisher Scientific, USA). After being rinsed in PBS, these cells were incubated for 1 h with a diluted secondary antibody (1:200 diluted goat anti-mouse IgG-FITC in 3% BSA, #A10530, Thermo Fisher Scientific, Waltham, MA, USA) in a dark room and washed with PBS. Subsequently, nuclear counterstaining was performed using Hoechst 33342 (Thermo Fisher Scientific, Waltham, MA, USA) (1:10,000 dilution) for 5 min. After the counterstaining, the images were obtained by fluorescence microscopy using Axio Observer.A1 (Carl Zeiss, Jena, Germany).

### 2.6. Transmission Electron Microscopy (TEM) Analysis

Prior to sacrifice, the implants were removed, and the cells were isolated with a cell scraper and fixed in Karnovsky’s solution. After sacrifice, the cells from the bony structures around the area where implants had been placed were collected and fixed in Karnovsky’s solution. They were washed in 0.1 M PBS 3 times every 15 min. The specimens were dehydrated through a graded 70–100% ethanol series, exchanged with propylene oxide, and embedded in a mixture of Epon 812 and Araldite. Ultrathin sections (70 nm) were cut using a Leica EM UC6 Ultramicrotome (Leica, Vienna, Austria). A ribbon of serial ultrathin sections from each bony specimen and implant were collected on copper grids and stained with uranyl acetate and lead citrate. The serial fields were photographed at ×500 magnification using a JEOL 1400-Flash electron microscope (JEOL, Tokyo, Japan) operated at 120 kV.

### 2.7. TEM Sample Preparation by Focused Ion Beam (FIB)

The implant specimens were fixed with Karnovsky’s solution and washed in 0.1 M PBS 3 times every 15 min. The specimens were dehydrated through a graded 70–100% ethanol series and finally treated with hexamethyldisilazane for 15 min. A Helios 650 (FEI, Hillsboro, OR, USA) dual-beam FIB system was used for the TEM sample preparation. The specimens were deposited with a platinum layer to protect the implant and bony surfaces prior to milling. A Ga^+^ ion beam accelerated voltage of 30 kV was used for milling. The TEM sample (under 100 nm) was attached to a Cu TEM grid. The TEM analysis at Cs-STEM was observed using a JEM-AFM200F (Cold FEG, JEOL, Tokyo, Japan).

## 3. Results

### 3.1. Parts of the Implant

The distribution of the main locations of cells was classified into three major structures within the implant: The root, the lower flank (LF), and the upper flank (UF). The implants used in this study were specially designed: The sharp V-shape parts for the firm engagement of bone, and the square area between the threads for the biologic response with no physical intervention such as stress (Figure 1A).

### 3.2. TEM Sample Preparation by Focused Ion Beam (FIB)

Surface characteristics along with cell attachment were probed using Cs-TEM from the FIB system. The cells were detected directly from an undecalcified specimen without the need to undergo cell isolation. The cells on the turned surface were unseen (Figure 1B), while on the SLA surface, organic matter was detected under the Pt-coated layer (Figure 1C).

### 3.3. Confocal Laser Scanning Microscopy (CLSM) Analysis of the Implant

The different topographical features [10,20,21] of the implants may affect cell attachment. Therefore, CLSM was used to measure the height parameters (S_a_), as well as the hybrid parameters (S_dr_), for the root, UF, and LF areas. The S_a_ values for the turned surface were 0.163 µm, 0.086 µm, and 0.098 µm, and the S_dr_ values were 10.3%, 8.2%, and 12.1% in the root, UF, and LF, respectively (Figure 1D). On the SLA surfaces, the S_a_ values were 1.14 µm, 1.17 µm, and 1.09 µm, and the S_dr_ values were 237%, 235%, and 239% for the root, UF, and LF, respectively. The S_a_ and S_dr_ values differed in terms of surface characteristics, with the SLA being higher; however, based on the different structural components, no differences were found in either the S_a_ or S_dr_. After the cells were fixed, the 3D topographical mapping of the cells also showed higher cell quantities in the root area of the SLA implants (Figure 1E). To see the correlation of the surrounding bone and the retrieved implant, the topographical parameters of the bone were also tested, but unfortunately, due to the sputtering of the Pt, the parameters could not be calculated in the bone area.

### 3.4. Scanning Electron Microscopy (SEM) Analysis of the Implant

**Figure 2:**
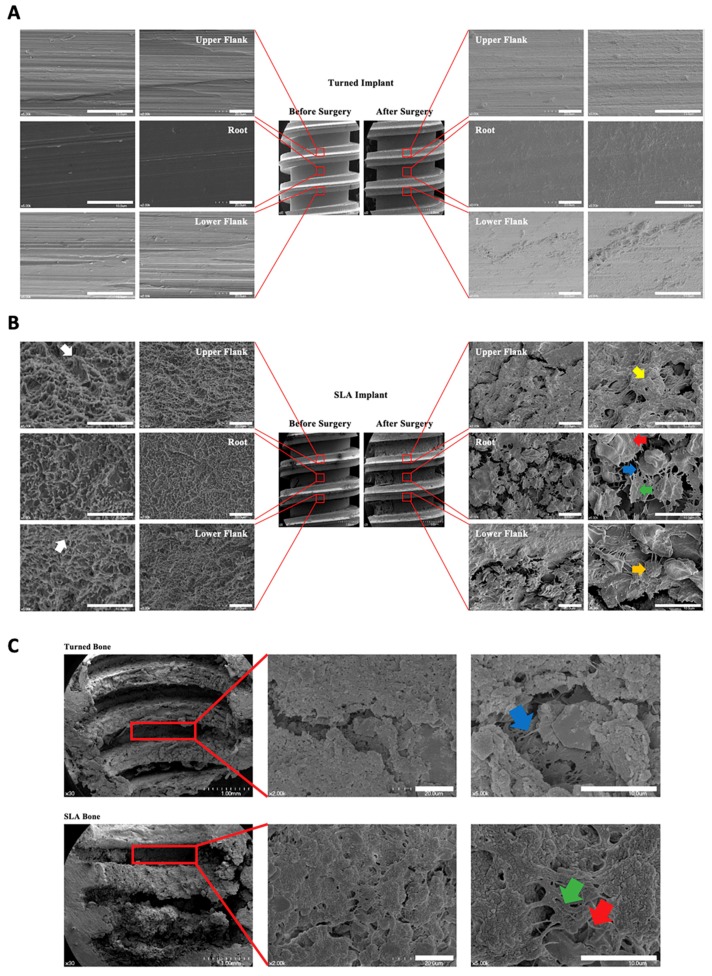
(**A**) Scanning Electron Microscopy (SEM) analysis of the turned implants. The smooth surface is displayed with many turned grooves. No cells were attached after 10 days. (**B**) SEM analysis of sandblasted, large-grit, and SLA implants. The surface characteristics of the SLA implants, which are typically porous, with honeycomb shapes (white arrow); the rather sharp peaks (left top white arrow) are definite, and the texture of the surface is rough. On the right-hand-side, the cells (green arrow) are shown to be mainly attached and actively spread out in the root area, with their filopodia extended (blue arrow). The fibrin can be seen on the cells (red arrow). In the UF, osteocytes and their processes are seen (yellow arrow). In the LF, the cell process is being extended, getting ready to migrate. The round cells are in static status (orange arrow). (**C**) SEM analysis of the surrounding bone of the removed turned and SLA implants at day 10. In the upper row, the overall image reveals traces of the smooth implant surface; thus, the bone texture is rather regular. The formation of the fibrin network is shown beneath some active cells (blue arrow). The lower row demonstrates the surrounding bone of the removed SLA implant. The texture of the bone is rather rough compared with that of the turned implant. The red blood cells can be seen underneath the cells. The mineralization grains (green arrow) are shown, and collagen (red arrow) is depicted well with striped bands. Scale bars = 10 μm.

In our research, the cell attachment and spreading varied depending on the structural differences in the implant thread. Cells were not attached in turned surfaces in all parts of the implants (Figure 2A). In the root area of SLA implants, an active cellular event took place. The cells were aggregated and spread out effectively, with their cellular processes extended. The fibrin of the cell could be detected. In the crest area, osteocytes and their processes were observed on both the UF and LF. However, they were not as active as the cells in the root area, in which the cells maintained a round shape, insufficiently spreading, and were in a static form (Figure 2B).

### 3.5. Scanning Electron Microscopy (SEM) Analysis of the Surrounding Bone of the Retrieved Implant

The SEM analysis of the surrounding bone of the retrieved turned implants revealed a fibrin network among the cells, whereas a striped pattern of supposed collagen bands was elucidated in the SLA implants. In the area where the bone was in contact with the UF and LF, a bone matrix was formed, while the crest area of the thread showed a fibrin network and an active cellular response. In the surrounding bone of the SLA-retrieved implants, the texture of the bone surface was rougher compared to that of the turned implants. Red blood cells were embedded, and a mineralization process had occurred in the crest area where the collagen bands were visible; cell folding could be observed with granules (Figure 2C).

### 3.6. Immunofluorescence Microscopy (IF) Analysis

**Figure 3:**
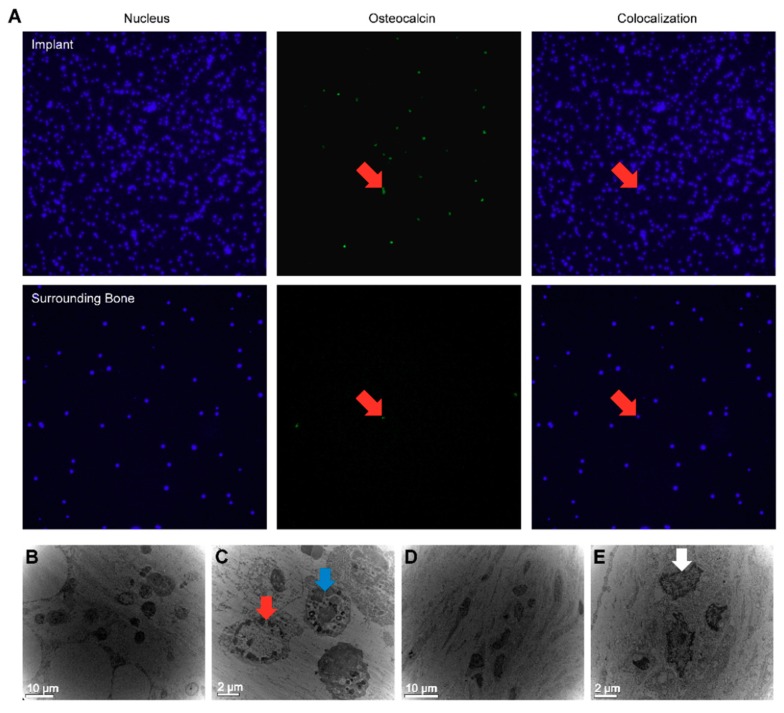
(**A**) Immunofluorescence microscopy (IF) of the SLA implants and surrounding bone on day 10, including nucleus, marker, and colocalization. Osteogenic cells (red arrow) are attached to the SLA implant surface rather than to the surrounding bone, which showed few osteogenic cells. The magnification of the photographs is ×200. (**B**) Transmission electron microscopy (TEM) analysis of retrieved SLA implant at day 10. (**C**) Macrophages (blue arrow) and osteogenic cell (red arrow) can be seen in the SLA implants. (**D**) TEM analysis of surrounding bone on day 10. (**E**) In the surrounding bone, osteocytes (white arrow) were detected.

Among the cells attached on the SLA surfaces, osteogenic cells needed to be identified because they are the key cells in bone formation. Accordingly, the attachment of osteogenic cells to the implant surface was confirmed through the use of osteocalcin—antibody targeted for rabbits in vivo. Immunofluorescence microscopy enabled the development of images of osteogenic cells on the SLA implants and the surrounding bone after 10 days, and consequently, confirmed the existence of osteogenic cells on the implant surface. While osteogenic cells were detected on the implant surface, the surrounding bone showed few osteogenic cells (Figure 3A).

### 3.7. Transmission Electron Microscopy (TEM) Analysis

The samples were also scrutinized by TEM. The TEM images of the SLA implants revealed macrophages and osteogenic cells (Figure 3B,C), while in the surrounding bone, osteocytes were detected (Figure 3D,E).

## 4. Discussion

In the present study, we aimed to determine the cell activity that occurs during the bone-forming process. We targeted the challenges concerning the lack of actual visualization of bone formation, and put much effort into presenting data on the active process in a bony environment with an actual 3D implant structure rather than the flat Ti discs used in in vitro studies. Our experimental data showed that a positive cell reaction occurred on the SLA surfaces, whereas the turned surfaces lacked cell adhesion. Meanwhile, the surrounding bone of the turned surface implants exhibited active cellular events. This may be confirmation of contact osteogenesis on the SLA surfaces. Whilst distance osteogenesis appears to occur around the smooth turned surfaces, it is well known that rougher turned surfaces (that were not investigated in this paper) also display contact osteogenesis [19,22,23,24].

According to the IF results seen, confirmation of osteogenic cells on the roughened implant surfaces might be further evidence of contact osteogenesis. However, further investigation is required to determine why only few osteogenic cells were detected on the bone surface—which is considered to be the place for distance osteogenesis. In addition, although limitations exist, in that cell classification is difficult in FIB specimens, the results reveal further evidence of contact osteogenesis on the SLA surface. Investigations are needed to better understand the link between such a phenomenon and the higher clinical long-term survival rate of implants with the SLA surfaces (over 95%), compared to that of turned implants (81–91%) [25,26].

The cells on the Ti implant surfaces seemed to be able to read the configuration of the structural parts of the implant. Considering the fact that implant geometry is a major factor in the initial stability of an implant inserted into bone and that osseointegration contributes to the subsequent stability, such SEM results imply that the initial, or primary, stability is associated with the shape of the crest area and that the secondary, or biological, stability is mainly connected to the cellular behavior at the root area [27,28].

Under the circumstances of immobility, exogenous foreign material such as Ti implants, exhibit bone demarcation instead of implant rejection; hence, the stability-enhancing structures of an implant may be of particular importance [29]. Cylindrical implants without threads have uniform but weak attachment to the bone, which is especially weak to shear stress. This weakness may have been one reason why the cylindrical implants displayed a lot of bone resorption in situ [30]. With regards to the electron microscopic images captured in the in vivo environment of this study, all the implant components shown, including the thread structure and microtopography, are important in the cellular response during the osseointegration process. Altering the surface roughness of a material may affect the biological processes regulating the behavioral mechanisms (e.g., cell activity, adhesion) of osteoblastic/immune cells, such extracellular protein deposition at the moment of implantation has an influence on the cellular behavior which later leads to differences in in vivo outcomes [31,32]. This study was qualitative. Quantitative investigations are necessary for various modified surfaces. Recently, implants made of other materials—including ceramic and polyetheretherketone (PEEK)—have been developed, the surfaces of which need to be further investigated with respect to this in vivo cellular response [33,34].

This study successfully presented direct evidence of the behavior of osteogenic cells on the implant surface in an in vivo environment at the electron microscopic level. According to the interpreted data herein, in the bone surrounding dental implants, cell behavior is determined by the treated surface of the implant, whereas cells attached to the SLA implants seem to be able to read the configuration of different implant structures and develop an attachment pattern that conforms to those structures.

## References

[1] Trindade R., Albrektsson T., Galli S., Prgomet Z., Tengvall P., Wennerberg A. (2018). Osseointegration and foreign body reaction: Titanium implants activate the immune system and suppress bone resorption during the first 4 weeks after implantation. Clin. Implant Dent. Relat. Res..

[2] Kohles S.S., Clark M.B., Brown C.A., Kenealy J.N. (2004). Direct assessment of profilometric roughness variability from typical implant surface types. Int. J. Oral Maxillofac. Implants.

[3] Yeo I.S., Han J.S., Yang J.H. (2008). Biomechanical and histomorphometric study of dental implants with different surface characteristics. J. Biomed. Mater. Res. B Appl. Biomater..

[4] Choi J.Y., Lee H.J., Jang J.U., Yeo I.S. (2012). Comparison between bioactive fluoride modified and bioinert anodically oxidized implant surfaces in early bone response using rabbit tibia model. Implant Dent..

[5] Kang H.K., Kim O.B., Min S.K., Jung S.Y., Jang D.H., Kwon T.K., Min B.M., Yeo I.S. (2013). The effect of the dltiddsywyri motif of the human laminin alpha2 chain on implant osseointegration. Biomaterials.

[6] Koh J.W., Kim Y.S., Yang J.H., Yeo I.S. (2013). Effects of a calcium phosphate-coated and anodized titanium surface on early bone response. Int. J. Oral Maxillofac. Implants.

[7] Kwon T.K., Lee H.J., Min S.K., Yeo I.S. (2012). Evaluation of early bone response to fluoride-modified and anodically oxidized titanium implants through continuous removal torque analysis. Implant Dent..

[8] Yeo I.S., Min S.K., Kang H.K., Kwon T.K., Jung S.Y., Min B.M. (2015). Identification of a bioactive core sequence from human laminin and its applicability to tissue engineering. Biomaterials.

[9] Wennerberg A., Albrektsson T., Chrcanovic B. (2018). Long-term clinical outcome of implants with different surface modifications. Eur. J. Oral Implantol..

[10] Wennerberg A., Albrektsson T. (2010). On implant surfaces: A review of current knowledge and opinions. Int. J. Oral Maxillofac. Implants.

[11] Choi J.Y., Jung U.W., Kim C.S., Jung S.M., Lee I.S., Choi S.H. (2013). Influence of nanocoated calcium phosphate on two different types of implant surfaces in different bone environment: An animal study. Clin. Oral Implants Res..

[12] Albrektsson T., Chrcanovic B., Molne J., Wennerberg A. (2018). Foreign body reactions, marginal bone loss and allergies in relation to titanium implants. Eur. J. Oral Implantol..

[13] Albrektsson T. (2018). On implant prosthodontics: One narrative, twelve voices-1. Int. J. Prosthodont..

[14] Choi J.Y., Kang S.H., Kim H.Y., Yeo I.L. (2018). Control variable implants improve interpretation of surface modification and implant design effects on early bone responses: An in vivo study. Int. J. Oral Maxillofac. Implants.

[15] Min S.K., Kang H.K., Jang D.H., Jung S.Y., Kim O.B., Min B.M., Yeo I.S. (2013). Titanium surface coating with a laminin-derived functional peptide promotes bone cell adhesion. Biomed. Res. Int..

[16] Araújo-Gomes N., Romero-Gavilán F., Sánchez-Pérez A.M., Gurruchaga M., Azkargorta M., Elortza F., Martinez-Ibañez M., Iloro I., Suay J., Goñi I. (2018). Characterization of serum proteins attached to distinct sol-gel hybrid surfaces. J. Biomed. Mater. Res. B Appl. Biomater..

[17] Kilkenny C., Browne W.J., Cuthi I., Emerson M., Altman D.G. (2012). Improving bioscience research reporting: The arrive guidelines for reporting animal research. Vet. Clin. Pathol..

[18] Trindade R., Albrektsson T., Galli S., Prgomet Z., Tengvall P., Wennerberg A. (2018). Bone immune response to materials, part i: Titanium, peek and copper in comparison to sham at 10 days in rabbit tibia. J. Clin. Med..

[19] Choi J.Y., Sim J.H., Yeo I.L. (2017). Characteristics of contact and distance osteogenesis around modified implant surfaces in rabbit tibiae. J. Periodontal Implant Sci..

[20] Wennerberg A., Albrektsson T. (2009). Effects of titanium surface topography on bone integration: A systematic review. Clin. Oral Implants Res..

[21] Yeo I.S. (2014). Reality of dental implant surface modification: A short literature review. Open Biomed. Eng. J..

[22] Albrektsson T., Brånemark P.I., Hansson H.A., Lindström J. (1981). Osseointegrated titanium implants. Requirements for ensuring a long-lasting, direct bone-to-implant anchorage in man. Acta Orthop. Scand..

[23] Davies J., Turner S., Sandy J.R. (1998). Distraction osteogenesis—A review. Br. Dent. J..

[24] Davies J.E. (1998). Mechanisms of endosseous integration. Int. J. Prosthodont..

[25] Buser D., Janner S.F., Wittneben J.G., Brägger U., Ramseier C.A., Salvi G.E. (2012). 10-year survival and success rates of 511 titanium implants with a sandblasted and acid-etched surface: A retrospective study in 303 partially edentulous patients. Clin. Implant Dent. Relat. Res..

[26] Adell R., Lekholm U., Rockler B., Brånemark P.I. (1981). A 15-year study of osseointegrated implants in the treatment of the edentulous jaw. Int. J. Oral Surg..

[27] Kwon T.K., Kim H.Y., Yang J.H., Wikesjö U.M., Lee J., Koo K.T., Yeo I.S. (2016). First-order mathematical correlation between damping and resonance frequency evaluating the bone-implant interface. Int. J. Oral Maxillofac. Implants.

[28] Meredith N. (1998). Assessment of implant stability as a prognostic determinant. Int. J. Prosthodont..

[29] Donath K., Laass M., Günzl H.J. (1992). The histopathology of different foreign-body reactions in oral soft tissue and bone tissue. Virchows Arch. A Pathol. Anat. Histopathol..

[30] Chrcanovic B.R., Albrektsson T., Wennerberg A. (2014). Reasons for failures of oral implants. J. Oral Rehabil..

[31] Romero-Gavilán F., Gomes N.C., Ródenas J., Sánchez A., Azkargorta M., Iloro I., Elortza F., García Arnáez I., Gurruchaga M., Goñi I. (2017). Proteome analysis of human serum proteins adsorbed onto different titanium surfaces used in dental implants. Biofouling.

[32] Dodo C.G., Senna P.M., Custodio W., Paes Leme A.F., Del Bel Cury A.A. (2013). Proteome analysis of the plasma protein layer adsorbed to a rough titanium surface. Biofouling.

[33] Bormann K.H., Gellrich N.C., Kniha H., Schild S., Weingart D., Gahlert M. (2018). A prospective clinical study to evaluate the performance of zirconium dioxide dental implants in single-tooth edentulous area: 3-year follow-up. BMC Oral Health.

[34] Najeeb S., Zafar M.S., Khurshid Z., Siddiqui F. (2016). Applications of polyetheretherketone (peek) in oral implantology and prosthodontics. J. Prosthodont. Res..

